# Angiogenic Properties of ‘Leukocyte- and Platelet-Rich Fibrin’

**DOI:** 10.1038/s41598-018-32936-8

**Published:** 2018-10-02

**Authors:** Jessica Ratajczak, Tim Vangansewinkel, Pascal Gervois, Greet Merckx, Petra Hilkens, Marc Quirynen, Ivo Lambrichts, Annelies Bronckaers

**Affiliations:** 1 Department of Morphology, Biomedical Research Institute, Faculty of Medicine and Life Sciences, Hasselt University, Diepenbeek, Belgium; 2 Department of Oral Health Sciences, Katholieke Universiteit Leuven (KUL) & Periodontology, University Hospitals Leuven, Leuven, Belgium

## Abstract

Leukocyte- and Platelet-Rich Fibrin (L-PRF) is an autologous platelet concentrate, consisting of a fibrin matrix enriched with platelets, leukocytes and a plethora of cytokines and growth factors. Since L-PRF is produced bedside from whole blood without the use of an anti-coagulant, it is becoming a popular adjuvant in regenerative medicine. While other types of platelet concentrates have been described to stimulate blood vessel formation, little is known about the angiogenic capacities of L-PRF. Therefore, this study aimed to fully characterize the angiogenic potential of L-PRF. With an antibody array, the growth factors released by L-PRF were determined and high levels of CXC chemokine receptor 2 (CXCR-2) ligands and epidermal growth factor (EGF) were found. L-PRF induced *in vitro* key steps of the angiogenic process: endothelial proliferation, migration and tube formation. In addition, we could clearly demonstrate that L-PRF is able to induce blood vessel formation *in vivo*, the chorioallantoic membrane assay. In conclusion, we could demonstrate the angiogenic capacity of L-PRF both *in vitro* and *in vivo*, underlying the clinical potential of this easy-to-use platelet concentrate.

## Introduction

Within the field of tissue engineering, establishing a vascular network is a key aspect in successfully regenerating damaged tissues^1,2^. A swift development of the vasculature supports cellular functions and survival by allowing exchange of nutrients, oxygen and waste products^2^. The use of biological products for wound treatment and surgical procedures has known an immense growth over the last two decades^2,3^. In particular the use of platelet concentrates, as a source of biomolecules involved in angiogenesis and wound healing, has gained a lot of attention due to their autologous nature and their cost-effectiveness^3,4^. The first preparation protocols involved two centrifugation steps and required biochemical handling^1^. Several improvements have been made since in order to develop a second-generation platelet concentrate, that can be produced without biochemical handling of the blood sample^1^. Leukocyte- and Platelet-Rich Fibrin (L-PRF) can be produced with one single centrifugation step (400 *g* – 12 min) and without the need for biochemical handling^5–7^. L-PRF consists of three different components, all of which can influence angiogenesis and wound healing. The white blood cells present in L-PRF, including neutrophils and macrophages, secrete pro-angiogenic molecules^8–11^. Platelets are known to release a plethora of growth factors (such as vascular endothelial cell growth factor (VEGF), Fibroblast growth factor-2 (FGF-2), Platelet-derived growth factor (PDGF) and cytokines upon degranulation^3,12^. Last but not least, the fibrin matrix also contributes to the angiogenic potential of L-PRF. By capturing the released biomolecules, the fibrin matrix ensures a progressive release of these molecules over time^13–15^. To date, numerous studies have investigated the angiogenic and regenerative potential of other platelet derivatives. For example, platelet rich plasma (PRP) has been described to enhance endothelial proliferation^16–18^, migration, and tube formation^19^. Moreover, PRP also improves wound healing in preclinical animal models^20,21^. So far only one report investigated the effect of platelet rich fibrin matrix (PRFM) on angiogenesis *in vitro*^22^. Roy *et al*. reported a slow and steady release of VEGF and the induction of endothelial cell mitogenesis. However, the PRFM used by Roy and colleagues was produced using trisodium citrate and calcium chloride^22^. The aim of the present study is to evaluate growth factor release of L-PRF and to determine its effect on endothelial cell proliferation, migration and tube formation *in vitro*. Finally, the capacity of L-PRF to induce blood vessel formation is tested in an *in vivo* setting.

## Results

### Characterization of the L-PRF secretome

**Figure 1:**
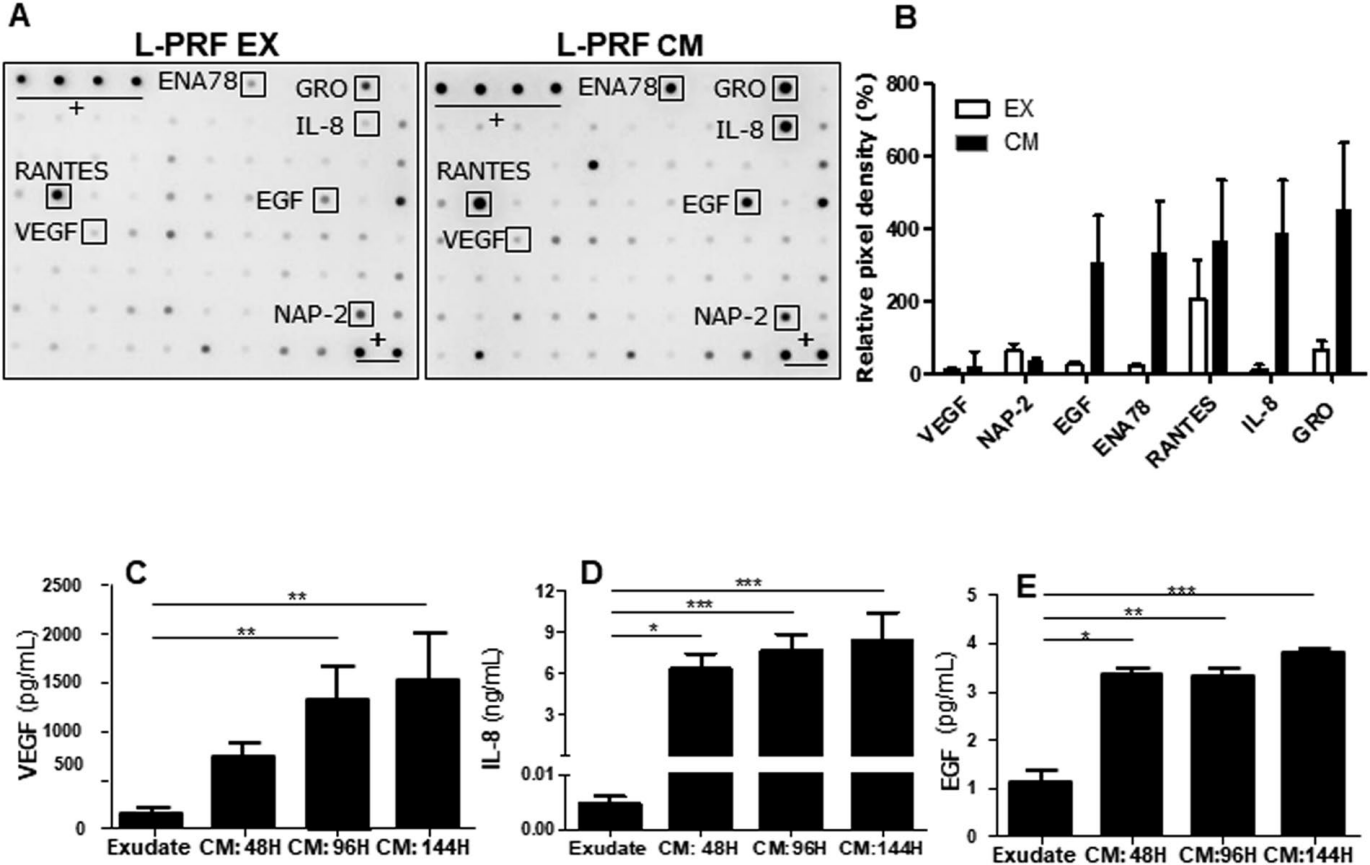
Protein release profile of L-PRF exudate (EX) and conditioned medium (CM). (**A**) An antibody array was performed to screen the proteins released from L-PRF EX and CM, representative picture of one donor (array was performed on 4 different donors, n = 4). (**B**) Relative pixel density was measured using ImageJ in order to compare relative protein levels between L-PRF EX and L-PRF CM. (**C–E**) In order to evaluate VEGF, IL-8 and EGF release over time, L-PRF clots were incubated in medium for 48 h, 96 h and 144 h before protein levels were measured with ELISA. L-PRF exudate (n = 8) contained only low amounts of VEGF, IL-8 and EGF compared to L-PRF CM. (**C**) VEGF contents increased with increasing time, however only a minimal increment was present between 96 H (n = 12) and 144 H (n = 8). (**D**) IL-8 levels were substantially lower in L-PRF EX (n = 8) compared to L-PRF CM. IL-8 concentrations displayed minor increments with increasing incubation times of the CM (E) whereas EGF levels in L-PRF CM remained stable over time. (+) positive control spots; ENA 78 = epithelial-derived neutrophil-activating peptide 78; EGF = epidermal growth factor; GRO = growth regulated oncogene; IL-8 = interleukin-8; NAP-2 = neutrophil-activating peptide-2; RANTES = regulated on activation, normal T cell expressed and secreted; VEGF = vascular endothelial growth factor. Data are expressed mean ± SEM. ***p-value < 0.001, **p-value < 0.01 and *p-value < 0.05.

The first part of this study focused on investigating the growth factor release from L-PRF. An antibody array was performed in order to obtain a more general screening of the growth factors that are released from L-PRF (Fig. 1A,B). The array was performed on exudate (EX) and conditioned medium (CM) from four different donors. Relative pixel density was determined with using ImageJ to compare relative protein levels between L-PRF EX and L-PRF CM (see Supplementary Table S1 and Fig. 1B). Analysis indicated high protein levels of epidermal growth factor (EGF) present in L-PRF CM compared to L-PRF EX. Furthermore, four other proteins: epithelial-derived neutrophil-activating peptide (ENA78), growth regulated oncogene (GRO), neutrophil-activating peptide-2 (NAP-2) and interleukin-8 (IL-8) were found to be abundantly present in L-PRF CM, whereas only minor levels of these proteins were detected in L-PRF EX. All four of these proteins are considered ligands to the IL-8 receptor β beta, also known as CXC chemokine receptor 2 (CXCR-2).

To validate the results of the antibody array screening tool, the levels of VEGF, EGF and IL-8 release from L-PRF at different time points were quantified by means of ELISA (Fig. 1C–E). VEGF, EGF and IL-8 levels in L-PRF exudate were significantly lower compared to protein levels in CM. VEGF levels increased with increasing incubation time, while IL-8 and EGF concentrations only showed a minor increase with increasing time. Since neither VEGF, nor IL-8 or EGF concentrations markedly increased after 96 hours, this time was chosen for harvesting L-PRF CM for all following experiments. After 96 hours the medium contained on average 1322 pg/mL VEGF, 7.7 ng/mL IL-8 and 3.3 pg/mL EGF.

### Functional analysis of the angiogenic potential of L-PRF *in vitro*

Angiogenesis is a tightly regulated biological process, which involves proliferation and migration of endothelial cells and finally tube formation. We investigated whether the factors released by L-PRF can affect endothelial cell behavior and blood vessel formation. Therefore, multiple *in vitro* assays were performed to mimic the different steps involved in angiogenesis.

**Figure 2:**
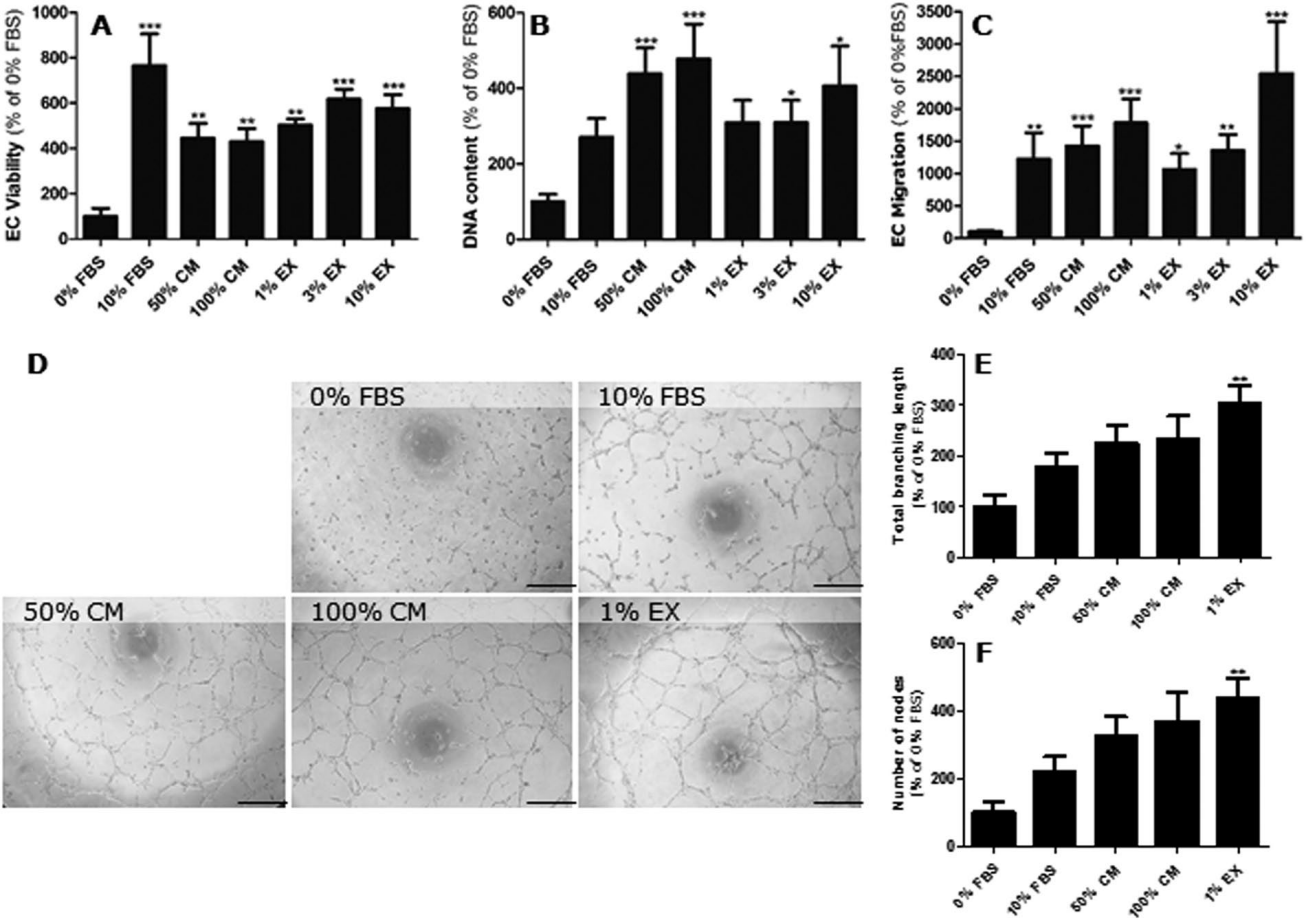
L-PRF enhances endothelial proliferation, migration and tubulogenesis. (**A**) L-PRF CM (n = 10) and L-PRF exudate (n = 8) enhance the metabolic activity of HUVEC as determined by MTT assay. (**B**) Incubating HUVEC with L-PRF CM (n = 9), 1% EX (n = 7), 3% EX (n = 9) and 10% EX (n = 7) resulted in increased proliferation, based on DNA content, compared to the negative control (0% FBS, n = 6). (**C**) L-PRF CM (n = 14) and L-PRF EX (n = 8) induce endothelial migration in the transwell migration assay. Both 50% CM as well as the addition of 100% L-PRF CM results in a significant increase in endothelial migration, compared to the negative control (0% FBS, n = 11). Every concentration (1–3–10%) of exudate that was tested, was able to induce endothelial migration, in a dose dependent manner. (**D**) Schematic overview of the tube formation experiment and representative images of endothelial tube formation after 6 hours of incubation with control medium (n = 5), L-PRF CM (n = 9) or L-PRF EX (n = 7). Incubation with L-PRF CM and L-PRF EX has a positive impact on endothelial tubulogenesis. Scale bar = 200 µM. (**E**) Incubating HUVEC with 1% L-PRF EX resulted in an increase in total branching length, whereas the increase in total branching length, caused by L-PRF CM was not significant. (**F**) The graph shows the average number of nodes, which was increased for all of the tested conditions except for the positive control. Data are represented as mean ± SEM. ***p-value < 0.001, **p-value < 0.01 and *p-value < 0.05 compared to 0% FBS. CM = conditioned medium; EC = endothelial cell; EX = exudate; FBS = fetal bovine serum; HUVEC = human umbilical vein endothelial cells.

One of the first steps in angiogenesis is endothelial cell proliferation. Hence, the effect of L-PRF on endothelial metabolic activity and proliferation was investigated by means of a 3-(4,5-Dimethylthiazol-2-yl)-2,5-diphenyltetrazolium bromide (MTT) assay and propidium iodide (PI) assay respectively (Fig. 2A,B). L-PRF CM and L-PRF EX significantly increased the metabolic activity of HUVEC compared to the negative control (Fig. 2A). Increasing the concentration of L-PRF CM or L-PRF EX did not further increase the metabolic activity.

Incubating HUVEC with L-PRF CM also resulted in a dose dependent increase in DNA content, and thus in the number of cells compared to the negative control (Fig. 2B). Exposure to L-PRF EX also resulted in a three- to four-fold increase in DNA content, and a significant increase in cell number was observed after incubation with 3% and 10% exudate. In contrast to the MTT assay, the effects of L-PRF CM and L-PRF EX did exceed the effect of 10% fetal bovine serum (FBS) with regard to DNA content (Fig. 2B).

In order to determine the capacity of L-PRF to induce endothelial migration, a transwell migration assay was performed (Fig. 2C). Both L-PRF CM and L-PRF EX caused a significant dose dependent increase in endothelial migration, compared to the negative control. A maximum response of endothelial cell migration was reached with 50% CM, with similar effects as the positive control. Migration towards L-PRF exudate showed to be dose dependent, with 10% exudate even exceeding the migratory response of the positive control containing 10% FBS.

Finally, the capacity of L-PRF to induce tubulogenesis was investigated using a Matrigel^TM^ tube formation assay (Fig. 2D–F). Despite the two-fold increase in total branching length when HUVEC were exposed to L-PRF CM, it was not statistically significant. However, we observed a significant increase in tubulogenesis with 1% L-PRF EX compared to the negative control. Addition of 10% L-PRF EX also induced tube formation but this led to the formation of tubes with aberrant forms, probably due to the high presence of growth factors. Moreover, incubation with either 50% or pure L-PRF CM resulted in a three-fold increase in the number of nodes whereas 1% L-PRF EX caused a four-fold increment.

### L-PRF induces blood vessel formation *in ovo*

A chicken chorioallantoic membrane (CAM) assay was performed to investigate whether L-PRF is also capable of inducing blood vessel formation *in vivo*. Following three days of incubation, a characteristic spooks wheel pattern of blood vessels was seen in every condition. Both L-PRF CM and L-PRF EX significantly increased blood vessel formation compared to the Matrigel^TM^ control condition.

**Figure 3:**
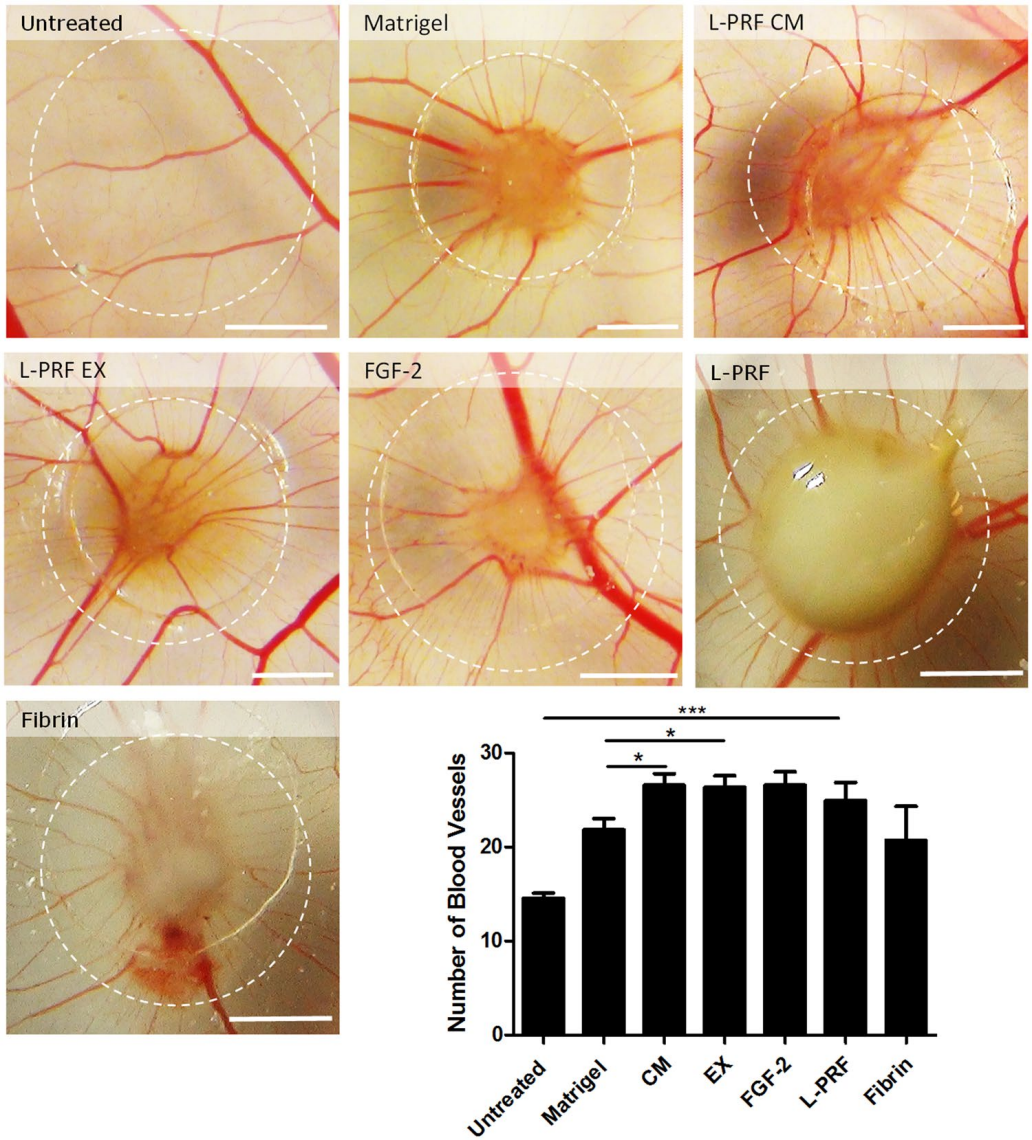
L-PRF induces blood vessel formation *in vivo*. Representative images of chorioallantoic membranes at E12. Untreated membranes (n = 21) were left completely untreated while others were incubated for 3 days with growth factor-reduced Matrigel^TM^ (n = 46) or with Matrigel^TM^ containing L-PRF CM (n = 57), L-PRF EX (n = 49), with L-PRF membrane discs (6 mm; n = 24) or with fibrin gel (n = 10) or 500 ng FGF-2 (n = 28). L-PRF CM and L-PRF EX increased the number of capillaries compared to Matrigel^TM^ and incubation with L-PRF membranes resulted in significantly more blood vessels compared to untreated membranes. Data are represented as mean ± SEM. *p-value < 0.05. CM = conditioned medium; EX = exudate; FGF-2 = fibroblast growth factor-2. Scale bar = 2 mm.

As a next step, we investigated whether the fibrin matrix itself could enhance blood vessel formation (apart from the growth factors released by L-PRF). Therefore, the eggs were incubated with 6 mm discs made from L-PRF membranes (Fig. 3). By using these membranes, the CAMs are not only exposed to the factors released from the L-PRF but also to the fibrin network. Incubation with L-PRF membranes resulted in a significant increase in the number of blood vessels, compared to untreated eggs. In order to determine the role of the fibrin matrix in the angiogenic capacities of L-PRF, eggs were treated with a fibrin gel, consisting of human fibrinogen and thrombin. The number of blood vessels in fibrin gel treated CAM’s was slightly higher than in control conditions, but this was not significant.

### The role of CXCR-2 and EGFR in L-PRF induced angiogenesis

Analysis of the L-PRF secretome revealed an abundancy of EGF and IL-8, particularly in L-PRF CM. As shown previously, both L-PRF CM and L-PRF EX were able to induce endothelial proliferation, migration and tube formation. In the current section we investigated whether these pro-angiogenic effects were mediated via the CXCR-2 and EGFR pathways by using a CXCR-2 antagonist (SB225002) and an inhibitor for the EGFR (AZD8931).

**Figure 4:**
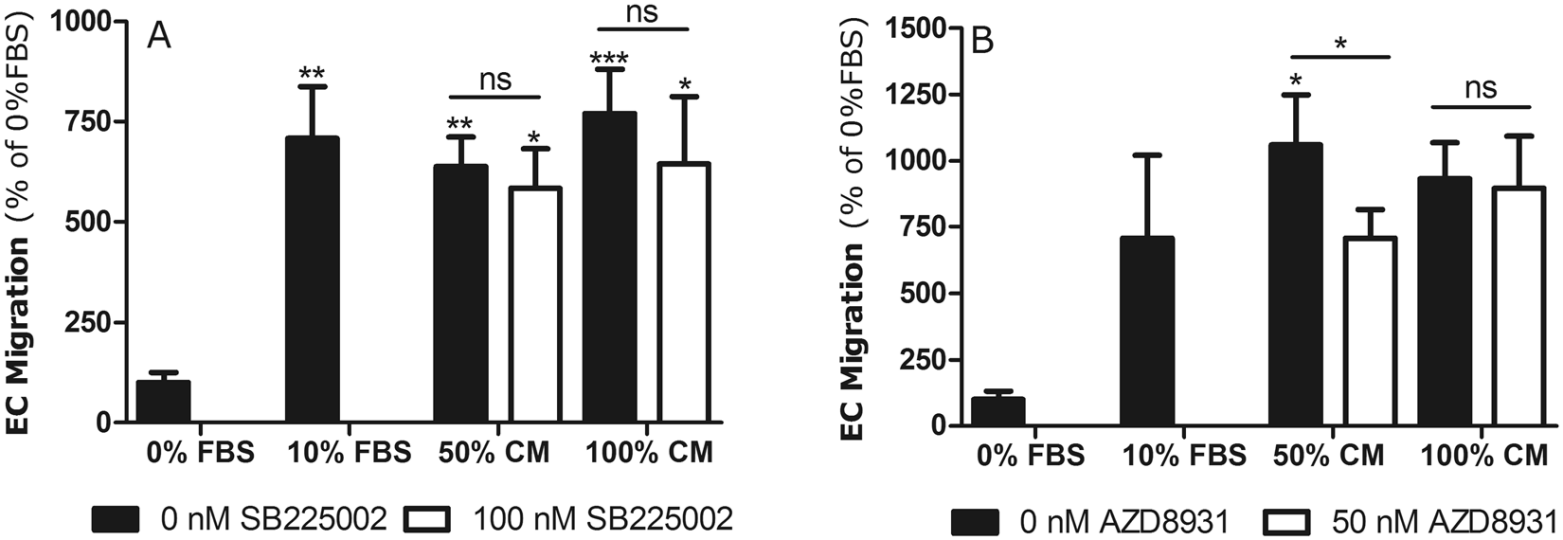
Endothelial migration towards L-PRF is partially mediated by the EGFR. (**A**) L-PRF CM induces endothelial migration with or without the addition of the CXCR-2 antagonist SB225002 (n = 8). (**B**) The migratory response of HUVEC towards 50% L-PRF CM (n = 6) can be diminished by adding 50 nM of AZD8931 to inhibit the EGFR. However, adding 50 nM of AZD8931 to 100% L-PRF CM did not reduce HUVEC migration. Data are represented as mean ± SEM. ***p-value < 0.001, **p-value < 0.01 and *p-value < 0.05. CM = conditioned medium; EC = endothelial cell; FBS = fetal bovine serum; HUVEC = human umbilical vein endothelial cells; L-PRF = Leukocyte- and Platelet-Rich Fibrin; ns = non-significant.

To examine whether L-PRF induces endothelial migration via the CXCR-2 pathway or the EGFR pathway, migration experiments were performed after pre-incubating HUVEC with inhibitors for these pathways (Fig. 4). Pre-incubation with SB225002 did not alter the endothelial migration towards L-PRF CM. In contrast, when HUVEC were pre-incubated with AZD8931 and allowed to migrate towards 50% L-PRF CM, the migratory response was significantly lowered (Fig. 4B). However, AZD8931 was not able to decrease endothelial migration towards 100% L-PRF CM. These data strongly suggest that the EGFR pathway is involved in endothelial migration induced by L-PRF.

## Discussion

Angiogenesis is indispensable for wound healing and tissue regeneration. However, impaired or deregulated angiogenesis can also contribute to ischemic diseases or chronic wounds such as in diabetes. In addition, for tissue engineering and bone healing angiogenesis is key to avoid apoptosis and necrosis of the implanted and newly-formed tissues^17,23^. Growth factors and other mediators released by activated platelets play an important role in tissue regeneration and revascularization. Platelet concentrates represent therefore a promising therapeutic tool in regenerative medicine^22^.

The L-PRF growth factor release was determined with an antibody assay and revealed an abundance of CXCR-2 ligands (IL-8, ENA-78, GRO and NAP-2) and EGF present in L-PRF CM and in lower levels in L-PRF EX. All these factors bind to CXCR-2, also known as IL-8 receptor beta, with high affinity^24–26^. These cytokines belong to the glutamic acid-leucine-arginine positive (ELR+) subfamily of CXC chemokines. ELR+ cytokines have been reported to have pro-angiogenic effects which are mediated via the CXCR-2 signaling pathway^27,28^.

Since an antibody array only indicates relative expression levels, the exact protein concentrations of VEGF, EGF and IL-8 released by L-PRF were quantified using ELISA. Significantly higher levels of these proteins were found in L-PRF CM compared to L-PRF EX. This rather large discrepancy can probably be ascribed to growth factor entrapment in the fibrin matrix. During centrifugation, polymerization of the L-PRF clot occurs slowly, creating a flexible fibrin network, which supports cytokine and growth factor enmeshment, resulting in a slow and gradual growth factor release^29–31^. Apart from protein entrapment, the production of VEGF by leukocytes could also partly account for the difference in VEGF levels between L-PRF CM and L-PRF EX^32^. Moreover, incubation of the L-PRF clot for 96 hours resulted in a two-fold increase in VEGF compared to the CM harvested after 48 hours. However, incubating L-PRF for an additional 48 hours, did not cause a further increase in VEGF levels. This is in contrast with other studies reporting a sustained VEGF release up to seven days^32,33^. However, in the study by Ehrenfest *et al*. VEGF release from compressed L-PRF membranes was evaluated, in contrast to the uncompressed L-PRF clots used in our study. Compression of the fibrin matrix could affect its architecture and therefore influence protein entrapment and release kinetics^34^. Furthermore, in these studies the medium was repeatedly renewed which further stimulates cytokine secretion since every medium change creates a cytokine-poor environment^33^.

While IL-8 levels in L-PRF exudate are in range with previous reports of IL-8 serum levels^35–37^, IL-8 concentrations were substantially higher in L-PRF CM. These high amounts of IL-8 in L-PRF CM are probably due to the induction of IL-8 production by fibrin. Fibrin has been described to induce IL-8 secretion in oral squamous cell carcinoma cells^38^, HUVEC^39^ and neutrophils^40^. Besides the ELR_+_ cytokines, the protein array also revealed the presence of relatively high quantities of EGF. Protein quantification via ELISA demonstrated a 5 times higher concentration of EGF in L-PRF CM compared to L-PRF EX. However, our results demonstrated only an average of 3.3 pg/mL EGF in L-PRF CM after 96 h, which is substantially less than the reported serum levels ranging from 200 pg/mL^41^ to ~1 ng/mL^42^ and even higher^43^. Bertrand-Duchesne *et al*. reported high levels of EGF (513 pg/mL) in human platelet rich plasma (PRP)^16^. The low levels detected in our study are probably due to the short half-life and the rapid cell diffusion of EGF^44^. Unfortunately, *in vitro* studies cannot account for the influence of the physiological environment on the behavior of the platelet concentrate with regard to cellular crosstalk and growth factor release^32^. However, *in vitro* characterization of these platelet concentrates remains an important step towards better understanding their effects *in vivo*.

Both L-PRF CM and L-PRF EX were shown to induce endothelial cell proliferation, migration and tube formation *in vitro*. This is in line with other reports, which have also demonstrated the ability of other human platelet concentrates to improve endothelial proliferation^16,18,45^, migration^32^ and tubulogenesis^19,45,46^. Although PRP has already been reported to induce endothelial tubulogenesis, to our knowledge this is the first study to report similar effects of L-PRF CM and L-PRF EX^19,45,46^.

In order to investigate whether the pro-angiogenic effects of L-PRF were mediated by the CXCR-2 or EGFR pathway, migration experiments were repeated with the addition of SB225002 (a selective CXCR-2 antagonist) or AZD8931 (a reversible inhibitor of EGFR). The addition of SB225002 to HUVEC did not influence endothelial migration towards L-PRF CM. However, addition of AZD8931 did cause a significant decrease in the migratory response to 50% L-PRF CM but not to 100% L-PRF CM. Possibly the used concentration of AZD8931 was not sufficient to inhibit EGF in the 100% CM condition. The non-peptide CXCR-2 inhibitor, SB225002 has been used in a variety of studies and has been reported to block IL-8 induced neutrophil migration^47^ and to possess antitumor activity^48,49^. Furthermore, Devapatla *et al*. showed a reduction in HUVEC survival, migration and tube formation when cells were exposed to 1 µM SB225002. However, SB225002 was more effective in combination with sorafenib, a VEGFR inhibitor^50^. AZD8931 has been shown to possess pro-apoptotic effects and inhibit xenograft growth in a range of cancer models^51^. Despite all these reports stating the efficacy of the inhibitors used in this study, these molecules did not successfully inhibit the pro-angiogenic effects of L-PRF in the current study. This might be caused by either unexpected side effects of the used blockers, but we hypothesize that the main reason for this is that multiple angiogenic factors work synergistically. Thus, when CXCR-2 and EGFR are blocked, the presence of other chemokines/growth factors compensate for this. The angiogenic capacity of L-PRF is possibly caused by multiple factors so that it can only be inhibited when two or more growth factors or general downstream signaling pathways are targeted. Therefore, it might also be worthwhile to investigate the involvement of more central downstream cellular signaling molecules such as ERK ½ or AKT in L-PRF-induced angiogenesis^52^. The mechanism of compensation after inhibition of a single angiogenic pathway has been repeatedly reported in cancer: bevacizumab, a potent inhibitor of VEGF, has been proven effective in animal studies but in clinical trials it failed to significantly improve the overall survival. Growth of tumor vessels depends not only on VEGF but also on various types of other angiogenic factors such as FGF-2 and angiopoietin-1. As a consequence, inhibition of only VEGF-related signals becomes compensated by other angiogenic factors, implying the importance of combinatorial treatments that target multiple pathways^53,54^.

In order to evaluate the ability of L-PRF to induce blood vessel formation *in vivo* a CAM assay was performed. Up until now this study has mainly been focusing on the angiogenic potential of the L-PRF secretome. However, growth factors are only one aspect of L-PRF that can be beneficial for blood vessel formation. It has also been demonstrated that the fibrin matrix can induce angiogenesis and guide the coverage of damaged tissues by influencing epithelial cells and fibroblasts^55^. Therefore, we included a fibrin gel derived from human fibrinogen and thrombin and intact L-PRF membranes, in order to combine the fibrin matrix with the platelet derived growth factors and to include the leukocytes. The human-derived fibrin gel was able to induce blood vessel formation *in vivo* which is in accordance with previous reports^56^. Incubating eggs with L-PRF membranes increased the number of blood vessels to a comparable level as L-PRF CM and L-PRF EX did. The increased angiogenesis cannot be ascribed to an inflammatory response as a reaction to the xenogeneic origin of L-PRF since the chick embryo lacks a mature immune system at this point in the development^57^.

In conclusion, this study demonstrates a strong pro-angiogenic effect of L-PRF *in vitro* and *in vivo*. Furthermore, we identified several important angiogenic molecules in L-PRF CM and EX such as IL-8 and EGF. Based on inhibition studies, these proteins together with other not yet identified factors, could be acting synergistically to induce angiogenesis. Pinpointing the key players of angiogenesis could aid in the search for biomarkers predicting L-PRF quality and possibly help with identifying patients benefitting the most from L-PRF treatment. The variety of preparation protocols and *in vitro* setups makes it difficult to compare results from different studies. This warrants standardization of preparation protocols in order to further characterize these platelet concentrates, which will hopefully lead to a better understanding of their *in vivo* effects. Despite these challenges our data suggest a promising role for platelet concentrates in the clinical setting of wound healing and tissue regeneration.

## Methods

### Preparation of Leukocyte- and Platelet-Rich Fibrin

Blood samples were obtained from 18 healthy donors with written informed consent. This study protocol and consent procedure were approved by the medical ethical committee from Hasselt University and the Clinical Trial Center from KU Leuven (S58789/B322201628215). All experiments were performed in accordance with relevant guidelines and regulations. Blood samples were collected in glass-coated plastic tubes (VACUETTE^®^ 9 mL Z Serum Clot Activator Tubes, Greiner Bio-One) by means of venipuncture, and centrifuged immediately (IntraSpin^TM^ Centrifuge, Intra-Lock, Boca Raton, Florida, USA) for 12 minutes at 2700 rpm (400 *g*).

For the preparation of conditioned medium (CM), L-PRF clots were incubated in 6 mL of serum-free α-MEM, supplemented with 2 mM L-glutamine (Sigma-Aldrich), 100 U/mL Penicillin (Sigma-Aldrich) and 100 μg/mL Streptomycin (Sigma-Aldrich). After 48 h, 96 h or 144 h the medium was collected, centrifuged for 6 minutes at 300 *g*, sterile filtered (Filtropur S0.2, Sarstedt, Nümbecht, Germany) and stored at −80 °C until further usage. To collect exudate (EX), L-PRF clots were transferred to a sterile box (Xpression™ Fabrication Box, Intra-Lock) and pressed into thin membranes, thereby releasing the exudate. The exudate was collected, filtered and stored at −80 °C until further usage.

### Human Cytokine Antibody Array

A Human Cytokine Antibody Array (Abcam, Cambridge, UK) was performed on L-PRF CM and exudate of two different donors at a protein concentration of 10 mg/mL, which was determined by the BCA method according to the manufacturer’s instructions (Thermo Scientific, Erembodegem, Belgium).

### Enzyme-Linked Immunosorbent Assay (ELISA)

EGF, interleukin-8 (IL-8) and VEGF were measured in L-PRF CM harvested after 48 h, 96 h and 144 h and in L-PRF exudate, using ELISA (Raybiotech, USA).

### Cell culture

Human umbilical vein endothelial cells (HUVEC) (HUVEC-2, 354151, BD) were cultured in endothelial cell growth medium (EBM-2, Lonza, Walkersville, MD, USA) supplemented with growth factors (EGM-2 SingleQuots™, Lonza) and 10% FBS and they were maintained at 37 °C, 5% CO_2_. Under these culturing conditions, HUVEC have a population time of 26.9 ± 4.5 hours (n = 8).

### 3-(4,5-Dimethylthiazol-2-yl)-2,5-diphenyltetrazolium bromide (MTT) – assay

In order to evaluate the effect of L-PRF on the metabolic activity of HUVEC, an MTT assay was performed as previously described^58^. Briefly, HUVEC were seeded (10,000 cells/well) in a 96 well plate in EGM-2 complete medium, and the next day the cells were washed and cultured in medium that contains different concentrations of L-PRF CM (50% and 100%) and L-PRF EX (1%, 3% and 10%) for 48 h. Afterwards, the medium was replaced by 500 μg/ml MTT (Sigma) in α-MEM. Following 4 h of incubation, the MTT solution was removed and a mixture of 0.01 M glycine in dimethylsulfoxide was added to dissolve the formed formazan. The absorbance was measured at a wavelength of 540 nm with a Benchmark microplate reader (Biorad Laboratories).

In addition, a control experiment was executed to test the viability of HUVEC under serum-deprived conditions (see supplementary information). For this experiment, an MTT assay was performed on HUVEC that were incubated in α-MEM supplemented with either 0, 1 or 10% FBS or EGM-2 complete medium for 24 h and 48 h, and the viability was compared with baseline levels (i.e. before replacing the medium at 0 h). The OD of the 0% α-MEM condition was not significantly reduced after 24 h and 48 h compared to baseline levels; whereas the OD of cells incubated in α-MEM 10% FBS or EGM-2 complete medium was significantly increased after 48 h (p < 0.001) (Supplementary Figure S1). Taken together, the data shown in Supplementary Figure S1 demonstrate that, within the time frame of 48 h, the viability of the cells is not negatively affected when they are cultured in medium with 0% serum. This indicates that this serum-deprived medium can be chosen as the basal medium from which CM can be prepared, and to which different concentrations of EX (1–3–10%) can be added (see section ‘Preparation of Leukocyte- and Platelet-Rich Fibrin’). More importantly, this implicates that the “α-MEM + 0% FBS” condition is an appropriate control in our experiments.

### Propidium iodide assay

HUVEC were incubated with different concentrations of L-PRF CM (50% and 100%) and L-PRF exudate (1%, 3%, 10%) for 48 h before medium was replaced by Lysis buffer A100 (ChemoMetec, Allerod, Denmark) and subsequently an equal amount of stabilization buffer B (ChemoMetec) supplemented with a propidium iodide (PI) solution (10 ug/mL, Sigma) was added. Cells were incubated for 15 minutes before measuring the fluorescent signal using the Fluostar Optima plate reader (BMG Labtech, Germany) at an excitation wavelength of 540 nm and an emission wavelength of 612 nm.

### Transwell migration assay

HUVEC migration towards L-PRF CM and exudate was evaluated by means of a transwell migration assay as described previously^58^. HUVEC were dissociated and labeled using a gentle cell dissociation reagent (STEMCELL technologies, Grenoble, France) supplemented with calceine acetoxymethyl (1.67 mM, BD). Fluorescence was measured using the Fluostar Optima plate reader (BMG Labtech) at an excitation wavelength of 485 nm and an emission wavelength of 520 nm. Medium with 0% serum served as a negative control, while medium with 10% FCS as a positive control (as this is also a blood derivative containing numerous angiogenic factors).

The CXCR-2 pathway was inhibited by adding 100 nM SB225002 (Selleckchem) to the HUVEC 15 minutes prior to seeding the cells in the culture inserts. The effect of EGF was inhibited by inhibiting the EGF receptor with 50 nM of AZD8931 (Selleckchem).

### Tube Formation

In order to examine the effect of L-PRF on endothelial tubulogenesis, a tube formation experiment was performed as previously described^59^. HUVEC (10 × 10^3^ cells/well) were cultivated in L-PRF CM (50%, 100%) or in L-PRF exudate (1%). After 6 h images were taken at a 4x magnification level with an inverted Nikon eclipse TS100 microscope equipped with a relay lens (Nikon Microscope DXM Relay Lens MQD42070) and a Jenoptik ProgRes C3 camera. The number of nodes and total branching length were determined using the Angiogenesis Analyzer plugin in Image J. Medium with 0% serum served as a negative control, while medium with 10% FCS as a positive control.

### Chorioallantoic membrane (CAM) assay

A CAM assay was performed as previously described^58^. Eggs with exposed CAM were incubated with a fibrin gel consisting of 20 mg/mL human fibrinogen (Merck, Darmstadt, Germany), 2.5 U/mL human thrombin (Merck) and 20 mM of CaCl_2_ (Sigma-Aldrich), L-PRF CM or L-PRF EX dissolved in growth factor-reduced Matrigel™ droplets (BD Biosciences) in a 1:1 ratio. CAMs to which droplets of 500 ng recombinant human FGF-2 (Immunotools) were added served as a positive control. Another group was treated with 6 mm discs created from L-PRF membranes using a biopsy punch (Stiefel, Middlesex, UK).

### Statistical Analysis

Statistical analyses were performed using Graphpad Prism software 5.03 (Graphpad Software, La Jolla, CA). Data normality was tested with D’Agostino & Pearson normality test. When Gaussian distribution was reached, experimental groups were compared using a one-way analysis of variance (ANOVA) with a Bonferroni post-test for groups ≤5. Non-parametric data were evaluated with a Kruskal-Wallis test combined with Dunn’s post-test. In the case of experiments involving the use of SB225002 of AZD8931, paired data were compared by a Friedman test followed by a Dunn’s multiple comparison post-hoc test for non-parametric data. When Gaussian distribution was reached, results were analyzed with Repeated Measures ANOVA with Bonferroni’s multiple comparison. Statistical significance was reached at p-values ≤ 0.05. All data were expressed as mean ± standard error of the mean (SEM).

## Electronic supplementary material

Supplementary Information

## Data Availability

All data generated or analysed during this study are included in this published article. The datasets generated during and/or analysed during the current study are available from the corresponding author on reasonable request.
