# Supplementary material for: Angiogenic Properties of ‘Leukocyte- and Platelet-Rich Fibrin’
Source: Sci Rep. 2018 Oct 2;8:14632. doi: 10.1038/s41598-018-32936-8 (PMC6168453; doi:10.1038/s41598-018-32936-8)
Supplement: Supplementary file 1 — Supplementary Information [file 41598_2018_32936_MOESM1_ESM.pdf]

Supplementary information

Angiogenic Properties of 'Leukocyte- and Platelet-Rich Fibrin'

Jessica Ratajczak[1], Tim Vangansewinkel[1], Pascal Gervois[1], Greet Merckx[1], Petra Hilkens[1], Marc Quirynen[2], Ivo Lambrichts[1], Annelies Bronckaers[1*]

[1] Department of Morphology, Biomedical Research Institute, Faculty of Medicine and Life Sciences, Hasselt University, Diepenbeek, Belgium

[2] Department of Oral Health Sciences, Katholieke Universiteit Leuven (KUL) & Periodontology, University Hospitals Leuven, Leuven, Belgium.

* Corresponding author

Supplementary Table S1: Quantitative analysis of the protein array (n = 4). Relative pixel density was measured using ImageJ in order to compare relative protein levels between L-PRF EX and L-PRF CM. Values represent the mean of the relative pixel density ± standard deviation.

	Exudate (EX)	Conditioned medium (CM)
**ENA-78**	21.99 ± 8.73	332.31 ± 289.64
**GCSF**	9.71 ± 15.69	1.37 ± 2.11
**GM-CSF**	15.30 ± 23.38	6.43 ± 7.75
**GRO**	64.67 ± 54.58	451.43 ± 373.07
**GRO-α**	8.00 ± 8.46	122.12 ± 150.48
**I-309**	1.70 ± 2.26	0.85 ± 1.65
**IL-1α**	1.55 ± 1.95	1.50 ± 3.00
**IL-1β**	9.50 ± 17.76	1.50 ± 2.48
**IL-2**	7.93 ± 14.84	0.80 ± 1.05
**IL-3**	9.68 ± 6.47	16.02 ± 26.46
**IL-4**	3.85 ± 6.06	6.18 ± 11.47
**IL-5**	1.22 ± 0.83	6.40 ± 11.15
**IL-6**	4.09 ± 5.35	20.99 ± 24.76
**IL-7**	9.13 ± 13.07	6.93 ± 7.69
**IL-8**	18.02 ± 22.69	387.32 ± 297.07
**IL-10**	10.49 ± 12.64	6.27 ± 7.40
**IL-12 p40/p70**	0.59 ± 1.19	0.85 ± 1.69
**IL-13**	0.47 ± 0.94	0.52 ± 1.04
**IL-15**	6.07 ± 9.22	0.57 ± 1.15
**IFN-γ**	3.39 ± 5.89	10.69 ± 19.88
**MCP-1**	19.77 ± 12.51	181.70 ± 175.33
**MCP-2**	4.56 ± 4.66	0.81 ± 1.06
**MCP-3**	1.92 ± 2.25	2.15 ± 2.72
**MCSF**	1.92 ± 2.26	1.65 ± 2.59
**MDC**	5.65 ± 4.05	3.02 ± 4.71
**MIG**	7.42 ± 8.02	3.43 ± 3.44
**MIP-1b**	14.96 ± 10.65	14.41 ± 10.50
**MIP-1δ**	6.25 ± 7.31	2.82 ± 4.66
**RANTES**	206.68 ± 216.36	366.24 ± 337.73
**SCF**	6.81 ± 10.40	8.39 ± 14.70
**SDF-1**	3.26 ± 5.95	19.39 ± 37.35
**TARC**	25.92 ± 23.80	18.98 ± 32.94
**TGF-β1**	3.85 ± 3.07	0.28 ± 0.33
**TNF-α**	3.72 ± 5.11	1.30 ± 1.57
**TNF-β**	5.06 ± 6.25	1.53 ± 2.21
**EGF**	26.99 ± 17.17	305.92 ± 264.18
**IGF-I**	9.34 ± 11.05	2.78 ± 4.15
**Angiogenin**	60.83 ± 25.59	71.16 ± 20.17
**Oncostatin M**	4.12 ± 4.79	2.60 ± 3.72

**Thrombopoietin**	5.07	±	8.63	16.69	±	31.25
**VEGF**	11.28	±	9.22	35.56	±	66.70
**PDGF-BB**	39.50	±	34.25	98.73	±	119.35
**Leptin**	22.73	±	15.31	7.96	±	5.35
**BDNF**	5.84	±	4.52	1.99	±	2.41
**BLC**	1.50	±	2.11	0.42	±	0.84
**Ck β 8-1**	3.56	±	4.52	1.86	±	2.77
**Eotaxin**	5.11	±	6.76	1.69	±	2.72
**Eotaxin-2**	6.96	±	6.77	4.08	±	5.25
**Eotaxin-3**	2.76	±	3.19	1.65	±	3.12
**FGF-4**	0.68	±	1.08	0.00	±	0.00
**FGF-6**	3.01	±	3.49	1.08	±	2.15
**FGF-7**	4.09	±	5.24	0.47	±	0.95
**FGF-9**	7.85	±	12.01	4.34	±	6.69
**Flt-3 Ligand**	0.80	±	0.94	0.05	±	0.09
**Fractalkine**	0.40	±	0.46	0.13	±	0.27
**GCP-2**	0.75	±	1.11	0.49	±	0.99
**GDNF**	3.50	±	4.54	2.04	±	2.92
**HGF**	1.85	±	1.87	1.15	±	2.22
**IGFBP-1**	4.34	±	5.36	2.79	±	3.32
**IGFBP-2**	17.31	±	5.04	11.55	±	9.63
**IGFBP-3**	5.63	±	6.51	2.68	±	3.12
**IGFBP-4**	4.96	±	4.67	0.71	±	1.42
**IL-16**	8.61	±	10.46	3.75	±	5.60
**IP-10**	9.18	±	6.43	3.05	±	3.53
**LIF**	12.23	±	14.19	3.65	±	4.23
**LIGHT**	1.41	±	1.75	0.54	±	1.00
**MCP-4**	1.25	±	1.83	0.39	±	0.77
**MIF**	3.49	±	5.06	3.44	±	4.03
**MIP-3α**	4.43	±	8.25	1.59	±	2.53
**NAP-2**	64.93	±	38.04	38.83	±	13.54
**NT-3**	11.07	±	12.84	3.41	±	4.04
**NT-4**	2.32	±	2.95	1.45	±	2.14
**Osteopontin**	10.38	±	7.66	13.70	±	16.83
**Osteoprotegrin**	4.28	±	3.99	1.21	±	1.41
**PARC**	3.15	±	3.66	1.94	±	2.36
**PIGF**	3.22	±	3.76	1.94	±	2.33
**TGF-β2**	23.57	±	9.80	8.20	±	8.14
**TGF-β3**	1.18	±	1.72	0.46	±	0.92
**TIMP-1**	11.38	±	11.84	8.55	±	9.88
**TIMP-2**	54.15	±	69.36	49.69	±	28.92

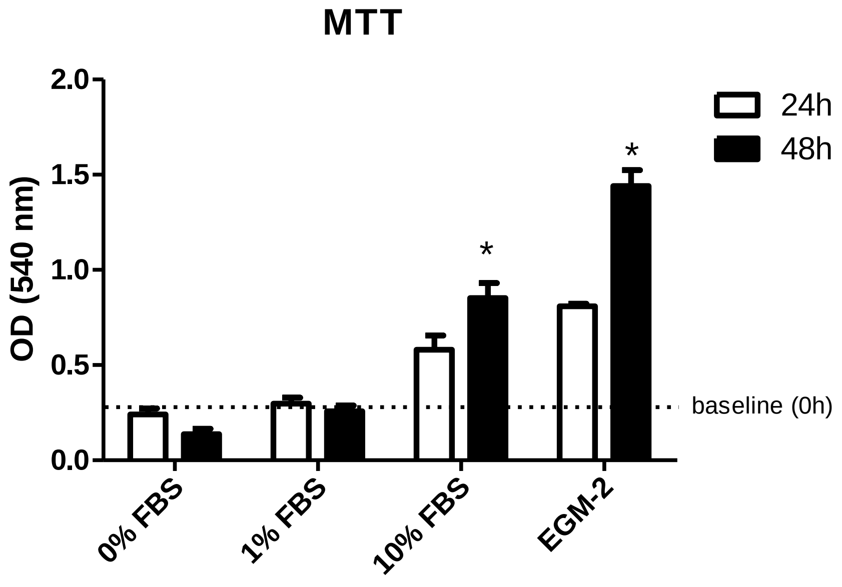

Supplementary Figure S1: Metabolic activity of HUVEC as determined by MTT assay. HUVEC were seeded in a 96-well plate and the next day, medium was replaced by α-MEM supplemented with either 0, 1 or 10% fetal bovine serum (FBS) or EGM-2 complete medium. An MTT assay was performed at baseline levels (just before replacing the medium at 0h), and after 24h and 48h. The optical density (OD) at baseline levels was 0.27 ± 0.15. The OD levels of 'α-MEM with 0% FBS' was not significantly reduced after 24h and 48h compared to baseline levels, indicating no significant reduction of viability. The OD of the conditions 'α-MEM 10% FBS' or 'EGM-2 complete medium' was significantly increased compared to baseline after 48h ($p < 0.001$). Data are presented as mean ± SEM. n = 5, *= p-value < 0.001 compared to baseline levels (0h) and compared to 'α-MEM + 0% FBS, 48h'.
